# Supplementary material for: The improved and the unimproved: Factors influencing sanitation and diarrhoea in a peri-urban settlement of Lusaka, Zambia
Source: PLoS One. 2020 May 13;15(5):e0232763. doi: 10.1371/journal.pone.0232763 (PMC7219762; doi:10.1371/journal.pone.0232763)
Supplement: S1 Appendix — Extracted sections: Sociodemographic data, sanitation, diarrhoea prevalence and chamber use. (PDF) [file pone.0232763.s001.pdf]

S1 Appendix: Adapted from the 'Household demographic and WASH questionnaire'

Reference ID: ______

**QUESTION**	**ANSWER**
Head of Household *(HH)*	◯ Yes<br>◯ No
**Part A: Household Head sociodemographic information**	
Year of birth *(e.g., 1986)*	______
Gender	◯ Male<br>◯ Female
Marital status	◯ Never married<br>◯ Married<br>◯ Living together<br>◯ Widowed<br>◯ Divorced
Education	◯ No education<br>◯ Primary<br>◯ Secondary<br>◯ More than secondary (Tertiary/Vocational)
Occupation	☐ Domestic worker (maid, gardener, baby sitter)<br>☐ Shop worker, General worker, Cleaner, Handyman, Office orderly, Security guard)<br>☐ Qualified clerk<br>☐ Government worker<br>☐ Private sector<br>☐ Self employed<br>☐ Student<br>☐ Unemployed<br>☐ Other (specify): ______
Monthly income	◯ No exact salary<br>◯ Less than K 525 per month<br>◯ Between K 525 and K 1,100 per month<br>◯ Between K 1,100 and K 2,200 per month<br>◯ Between K 2,200 and K 3,000 per month<br>◯ Above K 3,000 per month
Who owns this house?	◯ Resident<br>◯ Rented house<br>◯ Parents / Family
Number of Household Members (HM):	______

QUESTION	ANSWER
**Part B: Sanitation**	
Do you have a toilet available to your household?	◯ Yes<br>◯ No
How many toilets?	______
**(Questions refer to main/primary toilet)**<br>How many households use the toilet?	______
In total, how many people use the toilet?	______
Who owns the toilet?	◯ Household<br>◯ Landlord<br>◯ Neighbour<br>◯ Shared ownership<br>◯ Nearby establishment<br>◯ Public<br>◯ I don't know
Who is responsible for cleaning the toilet?	◯ Household<br>◯ Landlord<br>◯ Neighbour<br>◯ Shared responsibility with owners<br>◯ Nearby establishment<br>◯ Public<br>◯ No one<br>◯ I don't know
Who is responsible for maintenance of the toilet? *(including emptying)*	◯ Household<br>◯ Landlord<br>◯ Neighbour<br>◯ Shared responsibility with owners<br>◯ Nearby establishment<br>◯ Public<br>◯ No one<br>◯ I don't know
How often is the toilet cleaned?	◯ Several times a day<br>◯ Daily<br>◯ Once in a week<br>◯ Twice in a week<br>◯ More than twice in a week<br>◯ Once in a month<br>◯ Never

QUESTION	ANSWER
Who is responsible for toilet hygiene supplies? *(E.g., toilet paper, toilet cleaning materials, handwashing station – if any)*	◯ Household<br>◯ Landlord<br>◯ Neighbour<br>◯ Shared responsibility with owners<br>◯ Nearby establishment<br>◯ Public<br>◯ No one<br>◯ I don't know

**Part C: Diarrhoea Prevalence**	
Has any household member had diarrhoea in the past 2 weeks? *(3 or more watery stools within 24 hours in the last 2 weeks)*	◯ Yes<br>◯ No

**Part D: Chamber Use**	
Do you use a chamber?	◯ Yes<br>◯ No

Answer types:

◯ Multiple choice, one answer

⌂ Multiple choice, multiple answers possible

_____ Response to be filled in
