# Supplementary material for: The improved and the unimproved: Factors influencing sanitation and diarrhoea in a peri-urban settlement of Lusaka, Zambia
Source: PLoS One. 2020 May 13;15(5):e0232763. doi: 10.1371/journal.pone.0232763 (PMC7219762; doi:10.1371/journal.pone.0232763)
Supplement: S2 Appendix — Based on WHO-UNICEF JMP WASH service level criteria. (PDF) [file pone.0232763.s002.pdf]

S2 Appendix: Water, Sanitation and Hygiene Checklist

(Based on Progress on Drinking Water, Sanitation and Hygiene: 2017 Update and SDG Baselines. Geneva: World Health Organization (WHO) and the United Nations Children's Fund (UNICEF), 2017.)

Reference ID: ______

**QUESTION**	**ANSWER**
[1]House location	GPS Coordinates *(latitude, longitude, altitude, accuracy)*
**Part A: Toilet**	
Open defecation *(Signs of faecal matter)*	⌂ In the house<br>⌂ Near the household (in the yard)<br>⌂ Outside the household<br>⌂ None
Presence/availability of a toilet	◯ Yes<br>◯ No
[2]Toilet location	◯ In the room (self-contained)<br>◯ In the house<br>◯ Outside the house<br>◯ Outside the yard
[1]Picture of toilet	Image *(if consented)*
Toilet type	◯ Flush/ pour flush to piped sewer systems<br>◯ Septic tanks or pit latrines<br>◯ Ventilated improved pit latrines<br>◯ Composting toilets<br>◯ Pit latrines with slabs<br>◯ Pit latrine without slab/ platform<br>◯ Hanging latrine<br>◯ Bucket latrine
Toilet structure	⌂ Smooth cleanable floor<br>⌂ Slab (in good condition)<br>⌂ Wall<br>⌂ Roof<br>⌂ Frame (for roofing structure)<br>⌂ Door (lockable)
Toilet in use	◯ Excreta inside the toilet (latrine)<br>◯ Water source available
Toilets defecation hole	◯ Presence of closing device<br>◯ Absence of closing device
Cleanliness of the toilet	⌂ No signs of excreta around the hole, on the wall, on the floor<br>⌂ No bad smell<br>⌂ No contact to animals/ flies

QUESTION	ANSWER
Faeces disposal site	◯ In situ<br>◯ Septic tank<br>◯ Emptying (through company)<br>◯ No emptying<br>◯ Ditch<br>◯ Other<br>◯ Closed pipe from toilet
Service Level (WHO, UNICEF JMP, 2017)<br>*Safely managed: Improved toilet, faecal management on site, emptied, or to sewer, not shared.*<br>*Basic: Improved toilet, not shared.*<br>*Limited: Improved toilets, shared by more than 1 household.*	◯ Safely managed<br>◯ Basic<br>◯ Limited<br>◯ Unimproved<br>◯ Open defecation
**Part A: Handwashing (HW)**	
[2]HW practice	☐ Presence of hand washing station on site<br>☐ Water is available<br>☐ Soap is available: bar, liquid, detergent, sanitizer, ash<br>☐ Station designated for handwashing
[1]Picture of HW station	Image *(if consented)*
Service Level (WHO, UNICEF JMP, 2017)<br>*Basic: HW station with soap & water*<br>*Limited: HW station without either soap and/or water*<br>*No facility: No HW station*	◯ Basic<br>◯ Limited<br>◯ No facility
**Part A: Drinking Water**	
[2]Water source	◯ Piped water<br>◯ Boreholes/ tube wells<br>◯ Protected dug wells<br>◯ Protected Springs<br>◯ Packaged/ delivered water<br>◯ Unprotected dug well/ unprotected spring<br>◯ River, dam, lake, pond, stream, canal/ irrigation canal
Name of water source *(if public source)*	
[1]Water source location	GPS coordinates *(latitude, longitude, altitude, accuracy)*
[1]Picture of water source	Image *(if consented)*
Collection time	◯ Over 30 minutes<br>◯ Under 30 minutes<br>◯ On-site

QUESTION	ANSWER
Service Level (WHO, UNICEF JMP, 2017)<br>[3]*Above basic: Improved water source, on site, water available when needed*<br>*Basic: Improved water source, collection time under 30 mins*<br>*Limited: Improved water source, collection time over 30 mins.*<br>*Unimproved: Shallow wells, not lined*	◯ Above basic<br>◯ Basic<br>◯ Limited<br>◯ Unimproved<br>◯ Surface water

Answer types:

- ◯ Multiple choice, one answer
- ⌂ Multiple choice, multiple answers possible
- ____ Response to be filled in

[1]For verification of data
[2]Where there was more than one facility, focus was on the primary/main facility
[3]No water sampling was conducted. As such, Safely Managed Service Level for Drinking Water could not be determined
