# Supplementary material for: The improved and the unimproved: Factors influencing sanitation and diarrhoea in a peri-urban settlement of Lusaka, Zambia
Source: PLoS One. 2020 May 13;15(5):e0232763. doi: 10.1371/journal.pone.0232763 (PMC7219762; doi:10.1371/journal.pone.0232763)
Supplement: S3 Appendix — Independent variables: Toilet facility, chamber use and diarrhoea prevalence. (PDF) [file pone.0232763.s003.pdf]

S3 Appendix: Bivariate Odds Ratio Results

Table 1: Odds Ratio (OR) for factors associated with improved toilet facility

Variable	OR (95% CI)		Variable	OR (95% CI)
**Age**			**Households using toilet**	
18-29yo	1		≤5 households	1
30's	1.54 (0.70-3.43)		≥6 households	0.62 (0.32-1.21)*
40's	2.24 (0.92-5.44)*		Persons using toilet	
≥50	2.06 (0.84-5.03)*		≤9 persons	1
Gender			≥10 persons	1.39 (0.75-2.58)
Male	1		Responsible: Toilet cleaning	
Female	0.79 (0.33-1.86)		Resident	1
Marital Status			External (Landlord/Other)	1.12 (0.60-2.07)
Married/Living together	1		**Toilet cleaning frequency**	
Single	0.74 (0.38-1.43)		Several times a day to Daily	1
Education			Several times a week to Never	0.40 (0.14-1.15)*
Secondary/above	1		**Toilet maintenance (+Emptying)**	
Primary/below	0.77 (0.41-1.42)		Yes	1
Employment			No	0.46 (0.18-1.15)*
Employed	1		Responsible: Toilet Hygiene	
Unemployed	0.70 (0.37-1.32)		Resident	1
**Income**			External (Landlord/Other)	0.82 (0.44-1.51)
Regular	1		**Drinking water**	
Irregular	0.12 (0.03-0.39)***		Improved	1
House Ownership			Unimproved	0.12 (0.05-0.28)***
Resident/Family	1		**Handwashing**	
Rental	0.83 (0.45-1.55)		Facility	1
Household Members			No Facility	0.11 (0.04-0.27)***
≤5 persons	1		**Chamber use**	
≥6 persons	0.74 (0.39-1.38)		Yes	1
**Toilet ownership**			No	2.53 (1.33-4.83)***
Resident	1		**Diarrhoea prevalence**	
External (Landlord/Other)	2.46 (1.26-4.80)***		Yes	1
**Private vs. Shared toilet**			No	0.33 (0.03-1.50)*
Private	1			
Shared	0.41 (0.16-1.03)*			

*P<.25; **P < .05; ***P < .01

Factors eligible for backwards stepwise regression **in bold**

Table 2: Logistic regression analysis of factors associated with using a Chamber

Variable	OR (95% CI)		Variable	OR (95% CI)
**Age**			**Households using toilet**	
18-29yo	1		≤5 households	1
30's	0.59 (0.26-1.33)*		≥6 households	1.90 (0.99-3.64)*
40's	0.70 (0.30-1.61)		Persons using toilet	
≥50	0.62 (0.26-1.46)		≤9 persons	1
**Gender**			≥10 persons	0.85 (0.47-1.55)
Male	1		**Responsible: Toilet cleaning**	
Female	2.21 (0.87-5.65)*		Resident	1
Marital Status			External (Landlord/Other)	1.82 (0.99-3.35)*
Married/Living together	1		Toilet cleaning frequency	
Single	0.91 (0.47-1.76)		Several times a day to Daily	1
Education			Several times a week to Never	1.20 (0.39-3.66)
Secondary/above	1		Toilet maintenance (+Emptying)	
Primary/below	1.35 (0.74-2.47)		Yes	1
Employment			No	0.52 (0.17-1.63)
Employed	1		**Responsible: Toilet Hygiene**	
Unemployed	1.42 (0.76-2.63)		Resident	1
Income			External (Landlord/Other)	2.29 (1.24-4.22)***
Regular	1		**Toilet facility**	
Irregular	1.37 (0.67-2.80)		Improved	1
House Ownership			Unimproved	2.53 (1.33-4.83)***
Resident/Family	1		Drinking water source	
Rental	1.22 (0.66-2.23)		Improved	1
**Household Members**			Unimproved	1.36 (0.61-3.05)
≤5 persons	1		Handwashing	
≥6 persons	1.49 (0.81-2.75)*		Facility	1
**Toilet ownership**			No Facility	1.21 (0.66-2.24)
Resident	1		**Diarrhoea prevalence**	
External (Landlord/Other)	0.35 (0.18-0.67)***		Yes	1
Private vs. Shared toilet			No	0.20 (0.07-0.56)***
Private	1			
Shared	0.63 (0.28-1.42)			

*P<.25; **P < .05; ***P < .01

Factors eligible for backwards stepwise regression **in bold**

Table 3: Logistic regression analysis of factors associated with any household member having Diarrhoea in the past 2 weeks

Variable	OR (95% CI)		Variable	OR (95% CI)
**Age**			**Households using toilet**	
18-29yo	1		≤5 households	1
30's	0.37 (0.09-1.50)*		≥6 households	2.04 (0.74-5.66)*
40's	0.45 (0.11-1.84)		**Persons using toilet**	
≥50	0.66 (0.18-2.40)		≤9 persons	1
**Gender**			≥10 persons	0.29 (0.09-0.94)**
Male	1		**Responsible: Toilet cleaning**	
Female	0.44 (0.14-1.34)*		Resident	1
Marital Status			External (Landlord/Other)	2.56 (0.87-7.55)*
Married/Living together	1		**Toilet cleaning frequency**	
Single	0.73 (0.23-2.32)		Several times a day to Daily	1
Education			Several times a week to Never	3.14 (0.79-12.46)*
Secondary/above	1		Toilet maintenance (+Emptying)	
Primary/below	0.55 (0.19-1.54)		Yes	1
Employment			No	1.19 (0.25-5.59)
Employed	1		**Responsible: Toilet Hygiene**	
Unemployed	1.36 (0.48-3.83)		Resident	1
Income			External (Landlord/Other)	3.04 (1.03-8.96)**
Regular	1		**Toilet facility**	
Irregular	0.80 (0.27-2.39)		Improved	1
House Ownership			Unimproved	0.33 (0.07-1.50)*
Resident/Family	1		Drinking water	
Rental	1.15 (0.42-3.16)		Improved	1
Household Members			Unimproved	1.22 (0.33-4.54)
≤5 persons	1		Handwashing	
≥6 persons	1.18 (0.43-3.24)		Facility	1
Toilet ownership			No Facility	0.76 (0.28-2.07)
Resident	1		**Chamber use**	
External (Landlord/Other)	0.82 (0.28-2.46)		Yes	1
**Private vs. Shared toilet**			No	0.20 (0.07-0.56)***
Private	1			
Shared	4.19 (0.54-32.56)*			

*P<.25; **P < .05; ***P < .01

Factors eligible for backwards stepwise regression **in bold**
