# Supplementary material for: The improved and the unimproved: Factors influencing sanitation and diarrhoea in a peri-urban settlement of Lusaka, Zambia
Source: PLoS One. 2020 May 13;15(5):e0232763. doi: 10.1371/journal.pone.0232763 (PMC7219762; doi:10.1371/journal.pone.0232763)
Supplement: Attachment — Submitted filename: Response to reviewers.docx [file pone.0232763.s004.docx]

**PLOS ONE Revision Feedback**

**Manuscript No. PONE-D-20-05682**

Title: The improved and the unimproved: Factors associated with peri-urban sanitation in Lusaka, Zambia

Feedback to Editor and Reviewer comments are as follows:

| **No.** | **Comment** | **Feedback** | **Page (P)/ Line (L)** |
| --- | --- | --- | --- |
|  | **Journal Requirements** | | |
| 1. | Please ensure that your manuscript meets PLOS ONE's style requirements, including those for file naming | Noted and edited:   - Vancouver style citations edited - Submitted files renamed | Throughout the document |
| 2. | Please note that the use of the word "slum" throughout the manuscript may be considered controversial and offensive. Please revise the manuscript to omit this word; this term can be substituted with other descriptors you have used such as "low income unplanned settlements" or "peri-urban settlements", which are appropriate. | Noted. Both instances have been edited. | Abstract: P.2 L27  Introduction: P.3 L52 |
| 3. | Additionally, you mention a questionnaire and WASH checklist in the text, but do not include these. Please upload them as supporting files, or alternatively you may include them within the main manuscript or via a link to an external website | Noted. Added as S1 & S2 Appendix  Cited in the manuscript | P.5 L94 |
|  |  |  |  |
|  | **Additional Editor Comments** | | |
| 1. | To be logical, please modify the title as: "The improved and the unimproved: Factors influencing sanitation and diarrhoea in a periurban slum of Lusaka, Zambia" | Noted and edited. The new title is:  The improved and the unimproved: Factors influencing sanitation and diarrhoea in a peri-urban settlement of Lusaka, Zambia | P.1 L3-4 |
| 2. | To check and correct the grammatical errors throughout and the citation style should follow the submission guidelines. | Noted and edited. | Throughout the document |
| 3. | Methodology:  - To add the study design and the study period. | Noted and added. | P.5 L91-92 |
| 4. | Methodology:  - To add the sample size determination | Since this data came from a larger study, the sampling information relating to the broader study has also been indicated. In addition, the confidence intervals at the 95% confidence level have been added. | P.7 L133-41 |
| 5. | Methodology: - To add the flow diagram for depicting the sampling procedure | Noted and added as Fig 1.  The flow diagram reveals 2 sampling methods: systematic random sampling for data collection and purposive sampling for sample eligible for data analysis.  Purposive sampling criteria: Access to toilet facility | Fig 1 citation:  P.5 L97-101  P.6 L104-7  P.7 L133-6 |
| 6. | Please consider condensing the conclusion section. There is a repetition in key findings and interpretation for instance:  LINES: 486-489 [Note: HTML markup is below. Please do not edit.] | Noted and edited. | P.28 L576-87 |
|  |  |  |  |
|  | **Reviewer #1** | | |
| 1. | General Comment 1:  Is the manuscript technically sound, and do the data support the conclusions?: Partly | Thank you for your feedback. I hope the edits made to the manuscript based on your insight improve its technical soundness and conclusions. |  |
| 2. | General Comment 2:  Has the statistical analysis been performed appropriately and rigorously?: No | Thank you for your insight. I have added the following:   - Data analysis process - Confidence interval for sample size - Citations for software packages used | - See Reviewer #1 points 4 & 5 - See Additional Editor Comments #4 |
| 3. | Line 92: In cases where household heads were absent who did you survey? | Data collectors either returned to the household at an alternative time to collect data from the household head, collected data from the eldest adult in the household, or skipped to the next house in the sequence.  Information has been added to the manuscript. | P.7 L147-9 |
| 4. | Line 168 : Add citation for data analysis software used | Noted and added. | P.11 L230-1 |
| 5. | Data analysis section needs elaboration for a better understanding (How was multivariate stepwise logistic regression computed? How was your logistic regression models constructed? | I have indicated the following information for  Multivariate stepwise logistic regression:  Odds ratio → Stepwise → Logistic Regression   - Odds ratio: variables with p <0.25 eligible for Stepwise. Indicated for each independent variable (see S3 Appendix) - Stepwise: Backwards. P = 0.1 - 0.25. Eligible variables included in logistical regression model - Logistic regression: p <0.05, CI: 95% | P.11 L232-53 |
| 6. | Line 218 : "being a private toilet" It should be "having a private toilet" | Noted and edited. | P.16 L301 |
|  |  |  |  |
|  | **Reviewer #2** | | |
| 1. | The manuscript is technically sound and the data supports the conclusions. The statistical analysis has been performed well. All data has been made available according to the PLOS data policy. To a very large extent, the manuscript is presented in an intelligible fashion and written in standard English. The article which is part of a larger study has been well described. It would however be useful if the authors gave a bit more information about the larger study to put the paper in context. | Noted and edited.  I have included more information about the larger study in the Introduction and Methodology. | Introduction  P.4 L67-70  Methodology  P.6 L104-12 |
| 2. | The research site was 1 of 2 informal settlements cited as epicentres of the 2017/2018 cholera outbreak in Lusaka. Within the settlement, 3 health zones were selected for data collection. How many health zones are within the settlement and, what informed the decision to select three health zones? | Noted and information added.  Please also refer to Fig 1: Flow diagram of sampling procedure. | Fig 1 citation:  P.5 L97-101  P.6 L106-12 |
| 3. | Kindly cite the ethics clearance certificate number for the 2 institutions as evidence of clearance | Noted and cited. | P.8 L159-61 |
| 4. | In cases where tenants lived in a cluster of houses with their landlords (a common occurrence in Lusaka peri-urban) only one household was targeted. What criteria was used to decide which household to target. | Noted and edited to clarify ‘sampling’ and ‘targeting’.  The 5^th^ house was targeted. If the 5^th^ house belonged to a cluster of households, the approached house was the first priority for sampling. Regardless, only one within the cluster was sampled. I have clarified this in the text. | P.7 L123-32 |
| 5. | Both sociodemographic and WASH data were collected using KoBoToolbox (questionnaire server) and Open Data Kit (phone application); and data collectors had 4 days training on how to use the application and fill in the questionnaires.  This gives the impression that the study was purely quantitative. I noticed however that under the section on household wash assessment, that, other data collection techniques such as Observation, short interviews and picture taking were used.   - The PI must mention the items looked out for during the observation and if this was done in every household or in the community as a whole. - The issues explored during the short interviews and the respondents of those short interviews must be specified. - The PI needs to indicate what pictures were taken and the purpose of the picture taking since nothing is said about the pictures taken in the results section. | Noted and clarified in the text. Data collected were via questionnaire and through observation:   - Observations were for the WASH checklist (see S2 Appendix) - Short interviews has been amended to questionnaire. It was directed at Household Heads (see S1 Appendix) - Photographs were taken purely for data verification purposes. | P.8 L169-79 |
| 6. | It is surprising to see so many young people (26%) and so many women (83.6 %) as household heads. Particularly when over 70% are living together. The PI acknowledges this. Some description of the context of the study area and possible reasons for this unusual findings would make things clearer for the reader. | Noted. I have added the definition of household head to the manuscript.  Possible reasons for these outcomes are:   - Higher poverty for females (more women living in low income communities) - Zambian population pyramid: Youthful, high mortality rate | P.18 L345-57 |
